# Educational inequalities in deaths of despair in 14 OECD countries: a cross-sectional observational study

**DOI:** 10.1136/jech-2024-222089

**Published:** 2024-07-16

**Authors:** Christopher Lübker, Fabrice Murtin

**Affiliations:** 1 Department of Health Sciences, University of York, York, UK; 2 Centre for Health Economics, University of York, York, UK; 3 Centre for Wellbeing, Inclusion, Sustainability, and Equal Opportunity, Organisation for Economic Co-operation and Development, Paris, France

**Keywords:** HEALTH EXPECTANCY, HEALTHCARE DISPARITIES, MORTALITY

## Abstract

**Background:**

Deaths of despair are a key contributor to stagnating life expectancy in the USA, especially among those without a university-level education, but these findings have not been compared internationally.

**Methods:**

Mortality and person-year population exposure data were collected in 14 Organisation for Economic Co-operation and Development member countries and stratified by age, sex, educational attainment and cause of death. The sample included 1.4 billion person-year observations from persons aged ≥25 years between 2013 and 2019. Country-specific and sex-specific contributions of deaths of despair to: (a) the life expectancy gap at age 25 and (b) rate differences in age-standardised mortality rates between high and low educational attainment groups were calculated.

**Results:**

Eliminating deaths of despair could reduce the life expectancy gap in the USA by 1.1 years for men and 0.6 years for women was second only to Korea, where it would reduce the gap by 3.4 years for men and 2.2 years for women. In Italy, Spain and Türkiye, eliminating deaths of despair would improve life expectancy gains by less than 0.1 years for women and 0.3 years for men, closing the educational gap by <1%. Findings were robust to controls for differences in population structures.

**Conclusions:**

Deaths of despair are a major determinant of educational inequalities in longevity in Korea and the USA, while having limited impact in Southern European countries, indicating substantial international variation and scope for improvement in high burden high-income countries.

**WHAT IS ALREADY KNOWN ON THIS TOPIC**

- Deaths of despair—deaths from drug overdose, suicide or alcoholic liver disease and cirrhosis—are a key contributor to stagnating life expectancy in the USA and increases in midlife mortality. These deaths have been found to occur disproportionally among people without a university-level education and therefore contribute to socioeconomic inequalities in health.

**WHAT THIS STUDY ADDS**

- To our knowledge, this study is the first to compare deaths of despair findings from the USA with other high-income countries, across 14 member countries of the Organisation of Economic Co-operation and Development (OECD) in 2013–2019. We found that the USA and Korea had the highest potential population life-year gain and reduction in educational inequalities from eliminating deaths of despair, while it would have a small impact in Southern European countries such as Italy, Spain and Türkiye.

**HOW THIS STUDY MIGHT AFFECT RESEARCH, PRACTICE OR POLICY**

- Tackling deaths of despair would benefit population longevity, reduce socioeconomic inequalities in health, and, since the majority occur in those under 65 years, could also generate economic via increased productivity. The findings in this study highlighted large variations in both the prevalence of and inequalities in deaths of despair across OECD countries, suggesting substantial scope for avoiding deaths of despair, especially in the USA and Korea.

## Introduction

 Deaths of despair comprise deaths from drug overdose (including alcohol poisoning), suicide and alcoholic liver disease and cirrhosis.^1^ Previous findings suggest deaths of despair are a key contributor to stagnating life expectancy in the USA, through increasing mortality rates among middle-aged populations without a university degree, especially among middle-aged white non-Hispanic populations.^2^

Reducing deaths of despair is a key challenge for public health policy, not only due to their effects on longevity—which is an essential component of well-being and therefore a policy concern in its own right—but also due to their contribution to health inequalities between the socially advantaged and disadvantaged. Moreover, since deaths of despair are mostly premature and avoidable deaths,^3 4^ reducing them may generate economic benefits.^5^

Studies on deaths of despair to date have focused primarily on the USA and there is a paucity of comparable cross-country evidence in high-income countries.^6^ International comparisons indicate whether existing findings are generalisable to other high-income countries or specific to the social, economic and health environment in the USA. International comparisons are also a hallmark of longevity and inequality research and policy because they may indicate the potential for improvement in outcomes of interest.^7^ This study aimed to address the current evidence gap, reporting the impact of deaths of despair on longevity and their contribution to educational inequalities in longevity in the USA and 13 other member countries of the Organisation for Economic Co-operation and Development (OECD).

## Methods

### Data

This study used anonymised country-level mortality and person-year population exposure from 14 OECD member countries between 2013 and 2019 by age, sex and education from persons aged at least 25 years from Murtin and Lübker.^8 9^ Each country-level dataset received ethical approval from the respective national statistical office and was shared with the OECD for this study.

Educational attainment was grouped according to the 2011 International Standard Classification of Education (ISCED-2011) into low (primary education and below, ISCED 0–2), medium (lower-secondary and upper-secondary, ISCED 3–4) and high (higher than upper-secondary, ISCED 5–8) categories.^10^ Seven countries (Australia, Canada, Spain, Italy, Lithuania, New Zealand and Sweden) reported data where death certificates were directly linked to administrative data containing educational qualifications from the deceased. Seven countries (Denmark, Hungary, South Korea, Poland, Slovakia, Türkiye and the USA) reported unlinked data, where educational attainment was reported at the time of death by relatives or public officials.

Cause of death was recorded according to the International Classification of Diseases, 10th Revision (ICD-10).^11^ Deaths were grouped into all-cause deaths and deaths of despair, comprising deaths by suicide (X60–X84 and Y87.0), alcohol-related deaths (E24.4, F10, G31.2, G62.1, G72.1, I42.6, K29.2, K70, K85.2, K86.0, O35.4, P04.3, Q86.0, R78.0, X45, Y15) and drug-related deaths (F11–16, X40–44, Y10–14) according to the Joint Economic Committee classification.^12^ A counterfactual dataset was also created, where deaths of despair were subtracted from all-cause deaths to illustrate the potential impact of eliminating deaths of despair on longevity and inequalities.

### Statistical methods

According to statistical guidance from the central European statistical office, Eurostat, missing education data were proportionally assigned according to the observed exposure in low, middle and high education groups, respectively, for each country-age-sex group.^9 13 14^ Country-specific, age-specific, sex-specific and education-specific mortality rates were corrected to: predict missing data points beyond country-specific age cut-offs; smooth the random variation mortality rates, which may result in volatile trends between age-groups and prevent implausible differences in mortality between education groups (see online supplemental appendix A for additional materials).

Period life expectancy was calculated using abridged life tables and Chiang’s method.^15^ Observations were pooled into 5-year age groups, decreasing the volatility of mortality rates, especially in higher age groups with lower exposure levels where small changes in the number of deaths can cause large changes in mortality rates. Life expectancy gaps were calculated as the differences between populations with high and low educational attainment. The Arriaga method was used to decompose the contribution of deaths of despair to the life expectancy gap across age groups, available in online supplemental appendix, figures B.1–B.3.^16^

Age-standardised mortality rates (ASMRs) were calculated using the 2010 OECD standard population to account for individual country-level variations in population structures over time and control for age as a confounder between the education–longevity relationship.^17^ To aid comparability with existing studies we introduced two age categories for ASMRs; ages 25–64 and 65–89, which distinguishes between decision-making between typical OECD pre-retirement and post-retirement age groups.^14^ Rate differences in ASMRs between high and low education groups were calculated for all-cause mortality, and the contribution of deaths of despair to the ASMR gap was reported using the same calculations. The ASMR slope and relative indices of inequality are available in online supplemental appendix 1, figure B.4.

The analyses were conducted in Stata V.17.0^18^ and R V.4.1.3.^19^

### Patient and public involvement

No members of the public were involved in the design or implementation of this study.

## Results

The sample consisted of 1 358 203 669 person-year observations for persons aged at least 25 years between 2013 and 2019, with 16 050 749 total deaths, of which 492 693 were deaths of despair. Thirty-two per cent of women in the sample had high educational attainment and 38% had low educational attainment, while 29% of the men in the same had high educational attainment and 39% had low educational attainment. For the seven countries that reported missing data, the proportions of observations with missing educational attainment were 1.3% for both the male and female samples (see online supplemental appendix table A.2).

### Impacts of deaths of despair on life expectancy

The mean life expectancy in the sample was 55.5 years for women and 53.3 years for men, with men standing to gain more from eliminating deaths of despair (0.6 years) than women (0.2 years), on average (see table 1). The USA would stand to gain the most years of life expectancy from eliminating deaths of despair; 0.6 years for women and 1.4 years for men, on average. The potential life expectancy gains of eliminating deaths of despair were also high for both women and men in Korea (0.4 years for women and 1.0 years for men) and Hungary (0.4 years for women and 1.1 years for men), while particularly high for men in Australia, Slovakia and Sweden (0.8 years), compared with women. Countries with low incidence of deaths of despair included Türkiye, Italy and Spain, where eliminating deaths of despair would add less than 0.1 years to female life expectancy and less than 0.3 years to male life expectancy.

**Table 1:** Life expectancy at age 25 and potential gains from eliminating deaths of despair by country, sex and education

Country	Females								Males							
	Low		Middle		High		All		Low		Middle		High		All	
	LE	-DoD	LE	-DoD	LE	-DoD	LE	-DoD	LE	-DoD	LE	-DoD	LE	-DoD	LE	-DoD
Australia	58.6	+0.5	61.4	+0.4	62.9	+0.2	60.4	+0.3	52.9	+1.4	57.3	+0.8	60.6	+0.4	56.6	+0.8
Cananda	57.4	+0.4	60.2	+0.3	62.5	+0.2	60.5	+0.3	52.9	+1.0	55.9	+0.8	59.5	+0.5	56.6	+0.6
Denmark	55.4	+0.4	58.7	+0.2	60.6	+0.2	58.1	+0.2	50.9	+0.7	54.4	+0.4	57.6	+0.3	54.3	+0.4
Hungary	50.5	+0.6	56.6	+0.4	58.6	+0.2	54.9	+0.4	40.1	+1.6	50.0	+1.1	55.3	+0.6	48.3	+1.1
Italy	60.3	+0.1	62.0	+0.1	62.7	+0.1	60.8	+0.1	55.4	+0.3	57.9	+0.2	59.5	+0.2	56.5	+0.2
Korea	52.4	+2.5	59.2	+0.6	60.3	+0.3	58.9	+0.4	42.5	+3.9	53.6	+1.4	57.1	+0.5	53.9	+1.0
Lithuania	51.2	+0.2	56.4	+0.2	59.6	+0.1	56.4	+0.1	41.6	+0.8	47.3	+0.7	53.5	+0.4	47.6	+0.6
New Zealand	57.6	+0.2	60.2	+0.2	61.6	+0.1	59.4	+0.2	54.3	+0.6	57.1	+0.4	59.4	+0.3	56.6	+0.4
Poland	55.0	+0.1	55.1	+0.1	58.2	+0.1	56.4	+0.1	44.4	+0.9	47.6	+0.4	54.9	+0.2	48.6	+0.4
Slovakia	50.0	+0.5	58.6	+0.3	63.6	+0.2	56.2	+0.3	38.3	+1.4	51.1	+0.9	55.4	+0.4	49.7	+0.8
Spain	60.8	+0.1	61.2	+0.1	64.3	+0.1	61.3	+0.1	55.0	+0.4	54.4	+0.4	60.9	+0.2	56.1	+0.3
Sweden	56.9	+0.7	59.5	+0.4	62.0	+0.2	59.5	+0.3	53.0	+1.5	56.3	+0.8	59.3	+0.4	56.1	+0.8
Türkiye	56.9	+0.0	60.0	+0.0	61.5	+0.1	57.3	+0.0	51.5	+0.1	54.1	+0.1	56.6	+0.1	52.4	+0.1
USA	54.2	+0.9	55.4	+0.8	59.1	+0.3	56.8	+0.6	48.5	+1.8	50.3	+1.7	56.8	+0.7	52.4	+1.4
Mean	55.5	+0.5	58.9	+0.3	61.3	+0.2	58.3	+0.2	48.7	+1.2	53.4	+0.7	57.6	+0.4	53.3	+0.6

Notes: Education is classified according to the 2011 International Standard Classification of Education (ISCED-2011) into low (primary education and below, ISCED 0–2), medium (lower-secondary and upper-secondary, ISCED 3–4), and high education (higher than upper-secondary, ISCED 5–8). Deaths of Ddespair: Ssuicide (X60-–X84, Y87.0), Aalcohol-Rrelated Ddeaths (E24.4, F10, G31.2, G62.1, G72.1, I42.6, K29.2, K70, K85.2, K86.0, O35.4, P04.3, Q86.0, R78, X45, Y15), and Ddrug-Rrelated Ddeaths (F11-–16, X40-–44, Y10-–14). Abbreviations: LE, life expectancy at age 25; -DoD, life expectancy gains (in years) from eliminating deaths of despair

-DoDlife expectancy gains (in years) from eliminating deaths of despairLElife expectancy at age 25

Eliminating deaths of despair would reduce the average life expectancy gap between high and low educational attainment groups by 0.4 years for women and 0.8 years for men, from 5.7 and 8.9 years, respectively. The USA showed the highest potential impact of eliminating deaths of despair in populations with high and middle educational attainment for both women (0.3–0.8 years) and men (0.7–1.7 years), while the potential life-year gains in Korea among populations with low educational attainment was the highest in the sample: 2.5 years for women and 3.9 years for men. Eliminating deaths of despair in Korea would reduce the life expectancy gap between populations with and high and low educational attainment by 2.2 years for women and 3.4 years for men, a 28% and 23% reduction in the absolute gap, respectively; the highest potential reduction in the sample.

**Figure 1:**
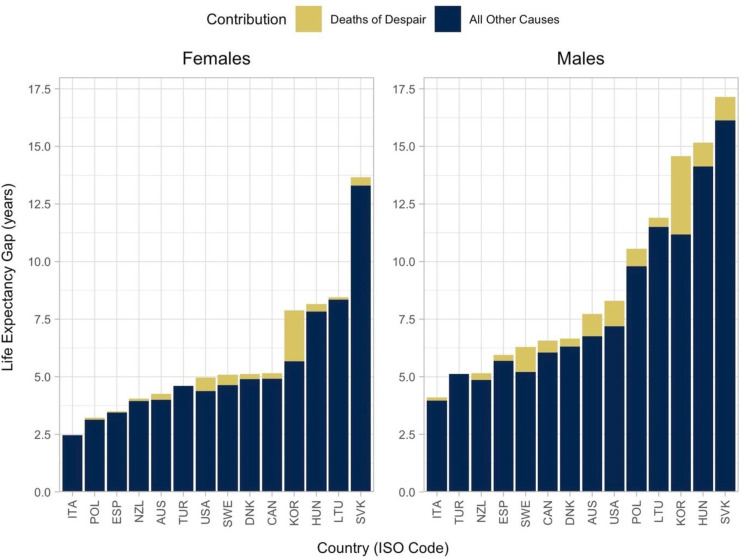
Contribution of deaths of despair to the life expectancy gap at age 25 between high and low educational attainment groups by country and sex. Notes: countries are reported in International Organisation for Standardisation three-letter codes. Education was classified according to the 2011 International Standard Classification of Education (ISCED-2011) into low (primary education and below, ISCED 0–2) and high education (higher than upper-secondary, ISCED 5–8). Deaths of despair (ICD-10 code): suicide (X60–X84, Y87.0), alcohol-related deaths (E24.4, F10, G31.2, G62.1, G72.1, I42.6, K29.2, K70, K85.2, K86.0, O35.4, P04.3, Q86.0, R78.0, **X45, Y15**) and drug-related deaths (F11–16, X40–44, Y10–14). This figure is replicated from Murtin and Lübker (2022) with permission.

In the USA, eliminating deaths of despair would reduce the life expectancy gap between populations with high and low educational attainment by 0.6 years for women and 1.1 years for men, a 12% and 14% reduction in the absolute gap, respectively (figure 1). Potential impacts on reducing inequality were also high for both women and men in Sweden, with a potential reduction by 0.5 years for women and 1.1 years for men, a 9% and 17% reduction in the absolute gap, respectively, and for men in Australia: 1.0 years reduction, or 13%. Meanwhile, eliminating deaths of despair would reduce the life expectancy gap in Italy, Türkiye and Spain by 1% or less.

The contributions of deaths of despair to the gap in life expectancy tended to be highest at younger ages and converge beyond age 65 for women and 75 for men (online supplemental appendix figure B.1–B.3). However, trends were not exclusively negative for age; for example, deaths of despair contributions rose with age for South Korea and Australia, peaking around age 35–39 before decreasing in all older age groups. Overall, deaths of despair among women and men aged 25–64 accounted for 7% and 11% of the total gap in life expectancy between high and low education groups on average, respectively. In ages 25–44, relative contributions to the total life expectancy gap between high and low education groups were highest in South Korea (27% and 21%, for women and men, respectively) and the US (10% for women and men).

### Deaths of despair mortality rates

**Figure 2:**
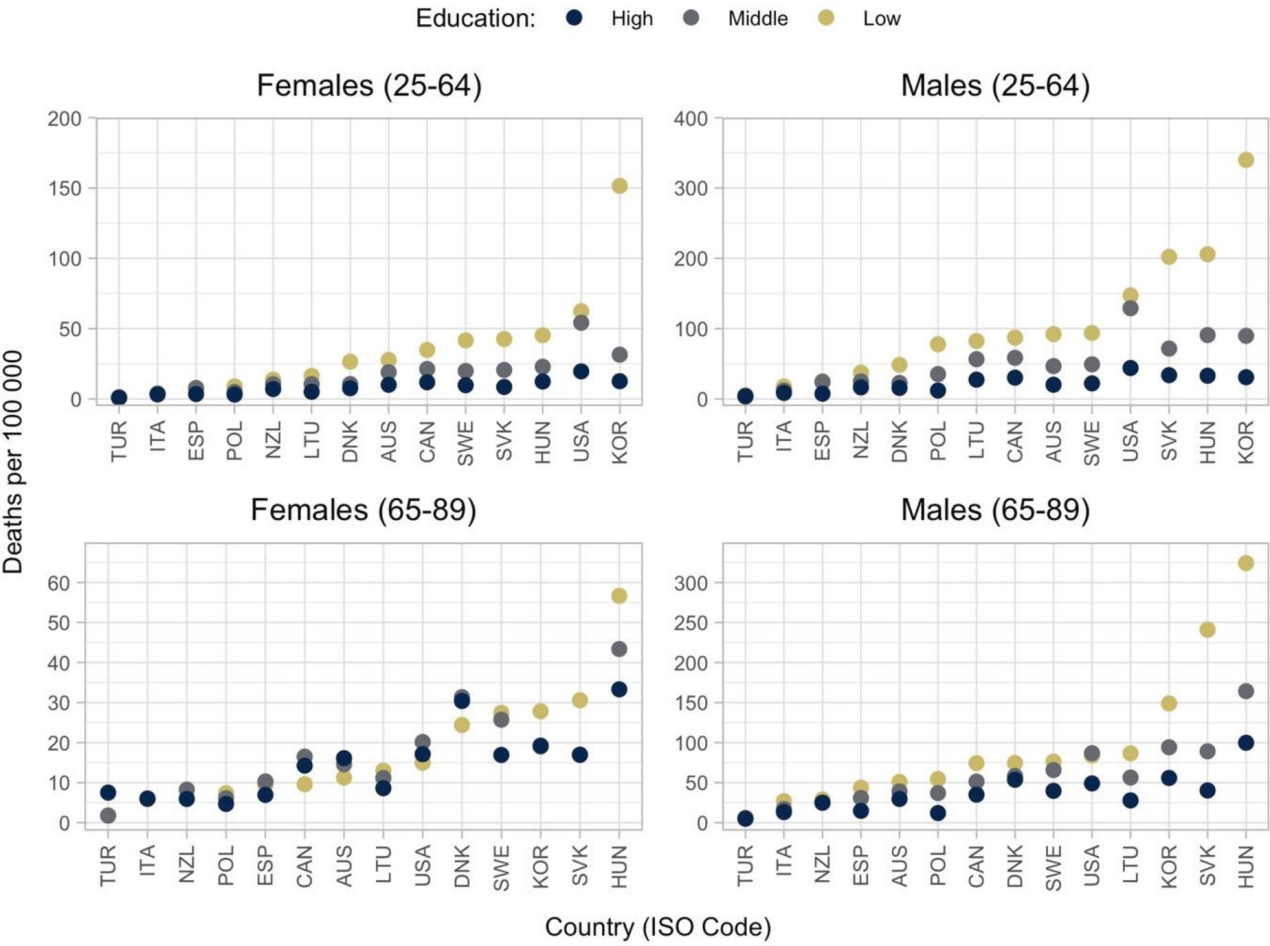
Age-standardised mortality rates from deaths of despair by country, sex, age-group and education. Notes: countries are reported in International Organisation for Standardisation three-letter codes. Education was classified according to the 2011 International Standard Classification of Education (ISCED-2011) into low (primary education and below, ISCED 0–2), medium (lower-secondary and upper-secondary, ISCED 3–4) and high education (higher than upper-secondary, ISCED 5–8). Mortality rates are standardised using the OECD 2010 standard population. Deaths of despair: suicide (X60–X84, Y87.0), alcohol-related deaths (E24.4, F10, G31.2, G62.1, G72.1, I42.6, K29.2, K70, K85.2, K86.0, O35.4, P04.3, Q86.0, R78.0, **X45, Y15**) and drug-related deaths (F11–16, X40-44, Y10–14). This figure is replicated from Murtin and Lübker (2022) with permission.

Figure 2 shows the ASMRs for deaths of despair, by age, sex and education. Among women and men aged 25–64 and among men aged 65–89, mortality rates were lowest among high education groups and highest among low education groups. The pattern was not clear among women aged 65–89, where reversed trends were observed in several countries, including Australia, Denmark, Canada and the USA. The largest mortality rates due to deaths of despair between ages 25 and 64 were recorded in Korea, the USA, Hungary and Slovakia. For women and men aged 25–64, the USA recorded the largest deaths of despair ASMRs among high and middle education groups (complete results are available in online supplemental appendix table B.1). Further evidence based on the slope and relative indices of inequality showed that, on average across countries, inequality in deaths of despair between educational groups decreases with age (see online supplemental appendix figure B.4 and table B.2). For women (men), the slope index of inequality decreased from 29 (89) to 5 (77) deaths per 100 000 from age 25–64 to 65–89.

**Figure 3:**
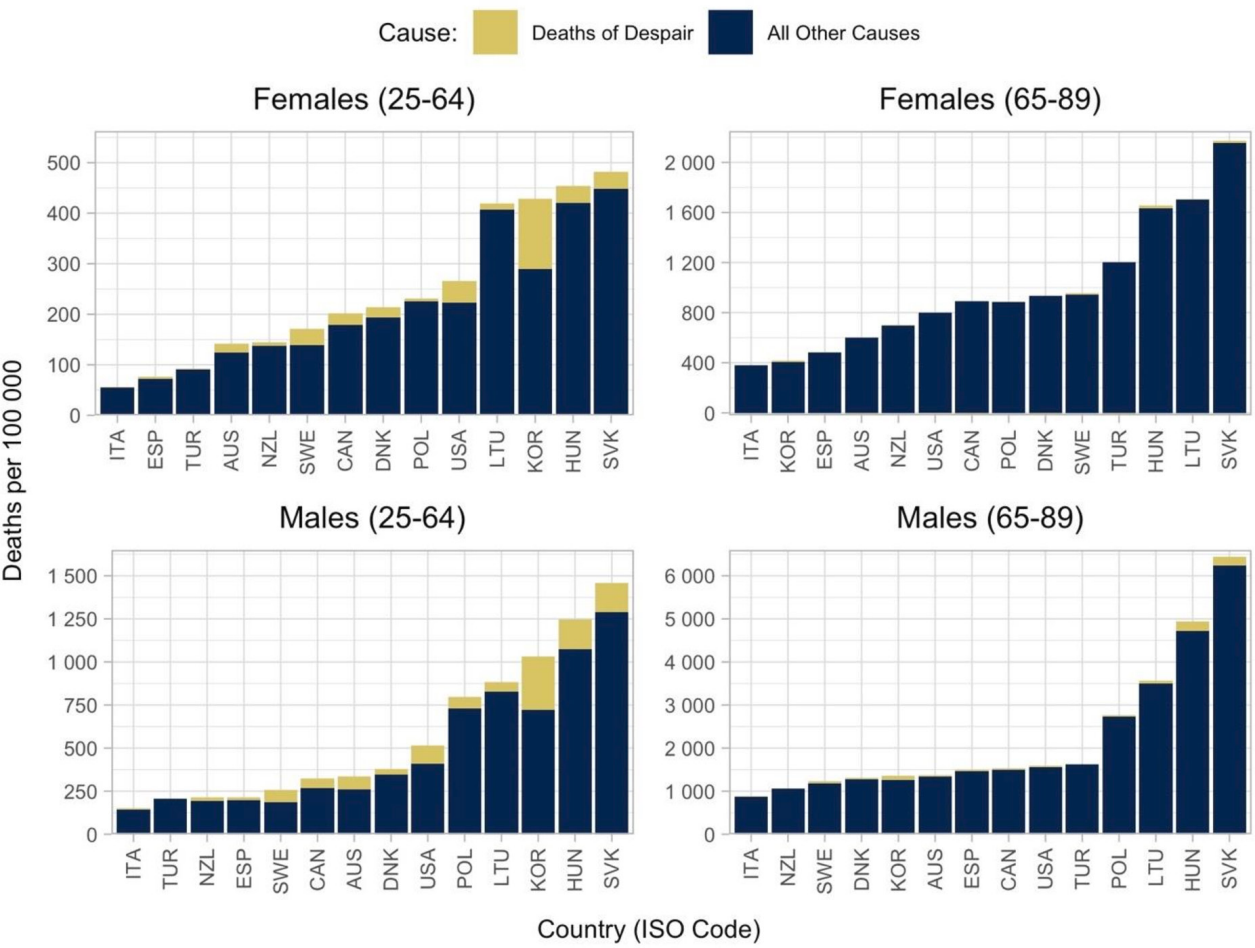
Contribution of deaths of despair to rate differences in age-standardised mortality rates between populations with high and low educational attainment by country, sex and age-group (deaths per 100 000). Notes: countries are reported in International Organisation for Standardisation three-letter codes. Education was classified according to the 2011 International Standard Classification of Education (ISCED-2011) into low (primary education and below, ISCED 0–2), medium (lower-secondary and upper-secondary, ISCED 3–4) and high education (higher than upper-secondary, ISCED 5–8). Mortality rates are standardised using the OECD 2010 standard population. Deaths of despair: suicide (X60–X84, Y87.0), alcohol-related deaths (E24.4, F10, G31.2, G62.1, G72.1, I42.6, K29.2, K70, K85.2, K86.0, O35.4, P04.3, Q86.0, R78.0, **X45, Y15**) and drug-related deaths (F11–16, X40–44, Y10–14). This figure is replicated from Murtin and Lübker (2022) with permission.

Figure 3 shows the contribution of deaths of despair to the difference in ASMRs across educational groups. Deaths of despair accounted for over 15% of the rate difference in ASMRs among women and men aged 25–64 in the USA, Korea and Sweden; and among men aged 25–64 in Australia and Canada. Deaths of despair contributed less than 5% (10%) of the total rate difference in ASMRs for women (men) in New Zealand, Spain, Lithuania, Poland, Italy and Türkiye. For women and men aged 65–89 deaths of despair contributions to the total ASMR rate difference were less than 2% for men in all countries except Canada, the USA, Slovakia, Sweden, Hungary and South Korea and were between 0 and −1% for women in Australia, Denmark, Canada, Türkiye and the USA (relative contributions are shown in online supplemental appendix figure B.5 to illustrate positive and negative contributions).

## Discussion

Across the 14 countries studied, eliminating deaths of despair would improve the average life expectancy by 0.2 years for women and 0.6 years for men. It would also reduce the average life expectancy gap between high and low educational attainment groups by 0.3 years for women and 0.8 years for men from 5.7 and 8.9 years, respectively. There was substantial between-country variation in the contribution of deaths of despair to health inequalities and longevity.

Eliminating deaths of despair would lead to the greatest life expectancy improvements in the USA (0.6 years for women and 1.1 years for men, on average), whereas the greatest reduction in the life expectancy gap would be seen in Korea; −2.2 years (−28%) for women and −3.4 years (−23%) for men. Deaths of despair also contributed substantially life expectancy gaps between education groups in Canada, Australia and Sweden, especially among men. Deaths of despair incidence was lower and more uniformly distributed across educational attainment in Southern European countries in the sample—Italy, Spain and Türkiye—where eliminating deaths of despair would close the educational gap by <1%. Findings were robust to controls for differences in population structures and age; deaths of despair accounted for over 15% of the rate difference in ASMRs among women and men aged 25–64 in the USA, South Korea and Sweden. Conversely, deaths of despair contributed less than 5% (10%) of the total rate difference in mortality rates for women (men) in New Zealand, Spain, Lithuania, Poland, Italy and Türkiye.

To our knowledge, there are no international studies comparing the US findings on socioeconomic inequalities in deaths of despair with other high-income countries using high quality data^6 20 21^ (while it is not possible to make direct comparisons due to differing years of data collection and deaths of despair classifications, Piñeiro *et al*^21^ found similar relative educational inequalities in deaths of despair for women in Spain, whereas inequalities in men were lower than in this study). This paper therefore provides a novel contribution to a subject of great concern,^22^ since deaths of despair have been identified as a driver of both health inequalities^2 23^ and stagnating life expectancy.^3 4^ Deaths of despair are also a subject of increasing concern because they have likely become more prevalent since the onset of the COVID-19 pandemic, which was associated with a 10%–60% increase in deaths of despair in 2020 in the USA.^24^

This study was subject to limitations. Due to the study design, causal interpretations are not possible from the analyses and more research is needed to untangle the complex pathways determining despair and subsequent deaths.^25^ Data quality varied between country-sources; unlinked and self-reported data may be subject to misreporting^26^ and phenomena such as ‘promoting the dead’ whereby the reported education level of the deceased is higher than the attained level, which could result in higher mortality reported in the high education group.^27^ Furthermore, subgrouping data by age, sex, education and cause of death may also lead to small sample sizes and volatile mortality rate estimates. We grouped ages into 5-year groups and pooled data across available years to minimise this problem. Where observation counts were especially small due to subgrouping, some data were censored by national statistical offices to protect anonymity of the deceased, which may influence results. This includes individual contributors to deaths of despair, which were not collected and the composite may obscure diverse underlying trends in drug overdoses, suicides and alcoholic liver disease and cirrhosis.^28^ While pooling data across available years minimises the problem of small sample sizes, it may also obscure trends in deaths of despair across the years with available data. We made simplifying assumptions to inform our data treatment, including (a) mortality rates generally rise log-linearly at all successive ages from age 30 and (b) education group mortality rates never cross over at ages beyond 85. Finally, countries did not all have data available for the same years, hence the sample years of the pooled data differ between countries.

Empirical studies suggest several evidence-based policies to reduce the incidence of deaths of despair, especially among the socially disadvantaged. Labour market policies, for example, have demonstrated reductions in suicide rates by reducing unemployment,^29^ providing income tax credits and raising the minimum wage.^30^ Policies targeting alcohol-related deaths^31^ may focus on heavy episodic (binge) drinking^32^ or areas with a high density of alcohol outlets.^33^ Minimum unit pricing has also been found to disproportionately benefit the health outcomes of the most disadvantaged.^34 35^ The role of healthcare policy for reducing drug-related deaths has been strongly emphasised,^36^ especially for prescription medicines, and the monitoring of drug prescriptions, in particular for opioids.^37^ Recent findings from the USA have also suggested that states where cannabis laws changes saw fewer drug-related deaths.^38^ These policies may have to balance the arguments of prohibition and safe supply, as well as the potential impacts across groups with varying social disadvantage.^39^

In the two countries that suffered the highest rates of deaths of despair, the USA and Korea, educational trends were inversed: having high educational attainment was particularly protective in the USA, whereas having low educational attainment in Korea was a particularly strong risk factor for deaths of despair. In other countries, the risk was more uniformly distributed, as the risk reduction was broadly similar when comparing high and middle education or middle and low education. Country-specific factors may partly explain the divergence observed between Korea and the USA, namely the opioid crisis in the USA^4^ and the suicide crisis in Korea^40^ (see online supplemental appendix figure B.6) (Online supplemental appendix figure B.6, for example, shows higher mortality rates among Korean women aged 25–44 than women aged 45–69 and stable mortality rates for Korean men aged 25–64, unlike other countries in the sample, showing an expected increase in mortality rates with age). Across all countries, however, the impact of deaths of despair was highest among younger age groups; generally, twice as large among those aged 25–44 years compared with those aged 45–64 years, suggesting greater need for intervention in younger adults with lower educational attainment.

Future research could further inform policy design by increasing country-coverage and continued monitoring deaths of despair. Where possible, these studies should use data where death certificates are directly linked to administrative data containing both educational attainment and other socioeconomic data of interest, such as income, occupation, race and ethnicity. Future studies should also include deaths of despair decomposed by individual causes to investigate the individual contributions from drug overdoses, suicides and alcoholic liver disease and cirrhosis, which would allow for better and more targeted policy recommendations. Furthermore, the cause of death classifications should be maintained across research to ensure comparability of results. Finally, where policies are implemented to tackle deaths of despair, their impact should be evaluated to document the average longevity effects, but also the distribution of health benefits across socioeconomic groups, to capture policy impacts on health inequalities.

## supplementary material

10.1136/jech-2024-222089 online supplemental file 1

## Data Availability

Data are available upon reasonable request. Data may be obtained from a third party and are not publicly available.
