# Supplementary material for: Educational inequalities in deaths of despair in 14 OECD countries: a cross-sectional observational study
Source: J Epidemiol Community Health. 2024 Jul 16;79(2):e222089. doi: 10.1136/jech-2024-222089 (PMC11874332; doi:10.1136/jech-2024-222089)
Supplement: online supplemental file 1 [file jech-79-2-s001.docx]

**Educational Inequalities in Deaths of Despair 14 OECD Countries: A Cross-Sectional Observational Study**

Appendix A. DATA ChaRACTERISTICS

Deaths of despair data were collected according to the relevant ICD-10 codes: Suicide (X60-X84, Y87.0), Alcohol-Related Deaths (E24.4, F10, G31.2, G62.1, G72.1, I42.6, K29.2, K70, K85.2, K86.0, O35.4, P04.3, Q86.0, R78.0, X45, Y15), and Drug-Related Deaths (F11-16, X40-44, Y10-14).

### A.1. Country-Source Assessments

This study is based on an analysis described in Lübker and Murtin (2022),^[[1]](#footnote-1)^ where overall data quality is assessed on a five-star scale for each country-source, five stars indicating the highest quality and one star the lowest quality data available for this analysis. For all sources, one star was subtracted for each of the following cases: (1) unlinked mortality data, (2) cause of death unavailable, (3) use of rolling population estimates, and (4) use of asymmetrical rolling population estimates, where mortality and population exposure data do not refer to the same year. For this study, we use only the highest quality data with three stars or more as primary sources for analyses (Table A.1) The combination of sources results in 14 countries covered by primary sources. For each country-source, we pool available data between 2013-19 to maximise sample sizes and smooth random variation in mortality rates.

Table A.1. Data overview

| Country | Source | Data | Primary | Death | Population | Analysis | Age | Linked | Cause of Death | Notes |
| --- | --- | --- | --- | --- | --- | --- | --- | --- | --- | --- |
|  |  | Quality | Source | Registers | Exposure | Period | Range |  |  |  |
| AUS | OECD | ***** | Yes | 2016 | 2016 | 2016 | 25-100+ | Yes | Yes | 1 |
| CAN | OECD | **** | Yes | 2012-16 | 2012-16 | 2013-16 | 25-90+ | Yes | Yes | 1-2 |
| DNK | OECD | **** | Yes | 2013-17 | 2013-17 | 2014-17 | 25-120 | No | Yes |  |
| ESP | OECD | **** | Yes | 2016-18 | 2016-18 | 2016-18 | 25-100 | Yes | Yes |  |
| HUN | OECD | **** | Yes | 2011-19 | 2016 | 2016-19 | 25-120 | No | Yes | 3 |
| ITA | OECD | ***** | Yes | 2012-16 | 2012-16 | 2013-16 | 25-120 | Yes | Yes |  |
| KOR | OECD | *** | Yes | 2015-18 | 2015 | 2015-18 | 25-84 | No | Yes | 3 |
| LTU | OECD | **** | Yes | 2015-19 | 2015-19 | 2015-19 | 25-120 | Yes | Yes |  |
| NZL | OECD | ***** | Yes | 2013-18 | 2013-18 | 2013-18 | 25-95+ | Yes | Yes | 1 |
| POL | OECD | **** | Yes | 2014-19 | 2011 | 2014-19 | 25-120 | No | Yes | 3 |
| SVK | OECD | **** | Yes | 2015-18 | 2015-18 | 2015-18 | 25-120 | No | Yes |  |
| SWE | OECD | ***** | Yes | 2015-18 | 2015-18 | 2015-18 | 25-120 | Yes | Yes |  |
| TUR | OECD | **** | Yes | 2014-15 | 2014-15 | 2014-15 | 25-100+ | No | Yes |  |
| USA | OECD | **** | Yes | 2018-19 | 2018-19 | 2018-19 | 25-85+ | No | Yes | 1 |

Note: (1) Data provided in 5-year age-groups; (2) Mortality data from CanCHEC are adjusted to reflect the structure of the Canadian population (Tjepkema et al 2019);^[[2]](#footnote-2)^ (3) In Hungary, Korea, and Poland, population exposure data were not available for all years with mortality data, so rolling population estimates are used to simulate the exposure in subsequent years (Mackenbach et al 2015).^[[3]](#footnote-3)^ Countries are reported in International Organization for Standardization (ISO) three-letter codes.

### A.2. Data treatments

Seven countries report missing education data. According to Eurostat guidance and the methodology of (Murtin et al., 2017_[1]_), missing education data are proportionally assigned according to the observed exposure in low, middle, and high education groups, respectively, for each country-age-sex group. Alternatively, all missing education data may be assigned to the low education category, on the assumption that missing education data are more likely to be attributable to low education individuals. We explore the impact of imputing all missing data to the low education group in a robustness exercise shown below.

In some instances, country-age-sex-education specific mortality rates were corrected to: (1) predict missing data points beyond country-specific age cut-offs; (2) smooth the random variation mortality rates, which may result in volatile trends between age-groups; and/or (3) prevent implausible cross-overs between education group mortality trends. Correction 1 ensures comparability across countries. Correction 2 standardises results according to Gompertz’s law, predicting that log mortality rates rise linearly from age 30 in each successive age group (Gompertz, 1825_[24]_). Correction 3 is imposed sparingly when small samples may spuriously lead to lower mortality rates for lower education groups than higher education groups, a pattern most commonly occurring above age 85.^[[4]](#footnote-4)^ In such cases, we impose a simple convergence rule, whereby the mortality rate of the low education group cannot fall below that of the middle education group, and the mortality rate of the middle education group cannot fall below that of the high education group. These corrections are applied for the sake of consistency and have small impacts on longevity estimates, since they occur at high age. We correct mortality rates by extrapolating log-linear trends:

$$\log m_{i,j,t}= a_{i,j}+b_{i,j}.t+\varepsilon_{i,j,t}$$

where m_i,j,t_ is the the mortality rate of sex-education-age group, *i*, *j*, and *t*, respectively. We use the five preceding five-year age-group data points to infer the predicted log mortality rates from a given age. Raw and predicted log mortality rate figures by age, sex, education, country, and source are available in Murtin and Lübker (2022) Appendix B and C, respectively.

US death registers use two types of education classification, either grade-based or degree-based. After discussion with OECD experts on the US education system, grades and degrees were allocated to low, medium or high educational attainment categories according to the mapping described in Table A4. In addition, Rostron et al. (2010) propose a correction factor of the number of deaths by educational level recorded in death registers, which is used in this paper and reported in Table A4. The Canadian data provided underreport mortality rates, since they exclude individuals living in institutions, collective housing, and the homeless (Tjepkema et al 2019). Corresponding average age-sex specific correction factors from 2011-16 were applied to reflect the Canadian population, assuming the same correction factor for each education group.

Table A.2. Distribution of educational attainment by country, source, and age for women

| **Country** | **Source** | **Total Person-Years** | **Age 25-44** | | | | | **Age 45-64** | | | | | **Age 65-84** | | | | | **Age 85+** | | | | |
| --- | --- | --- | --- | --- | --- | --- | --- | --- | --- | --- | --- | --- | --- | --- | --- | --- | --- | --- | --- | --- | --- | --- |
|  |  | **(1,000,000s)** | **Person-Years** | **Low** | **Middle** | **High** | **Missing** | **Person-Years** | **Low** | **Middle** | **High** | **Missing** | **Person-Years** | **Low** | **Middle** | **High** | **Missing** | **Person-Years** | **Low** | **Middle** | **High** | **Missing** |
|  |  |  | **(1000s)** | **(%)** | **(%)** | **(%)** | **(%)** | **(1000s)** | **(%)** | **(%)** | **(%)** | **(%)** | **(1000s)** | **(%)** | **(%)** | **(%)** | **(%)** | **(1000s)** | **(%)** | **(%)** | **(%)** | **(%)** |
| AUS | OECD | 8 | 3,413 | 11.8 | 49 | 29 | 10.2 | 2,913 | 22 | 47.1 | 20.7 | 10.2 | 1,538 | 30.8 | 41 | 13.7 | 14.4 | 179 | 35.6 | 31.9 | 8 | 24.5 |
| CAN | OECD | 42.8 | 13,828 | 10.7 | 38.9 | 50.4 |  | 18,970 | 14.8 | 41.3 | 43.9 |  | 9,120 | 25.8 | 38.6 | 35.5 |  | 928 | 40.5 | 33.9 | 25.6 |  |
| DNK | OECD | 9.7 | 3,580 | 19.1 | 43.3 | 34.6 | 3 | 3,767 | 23.4 | 47.5 | 27.1 | 2 | 2,197 | 31.3 | 43.7 | 22.9 | 2.1 | 192 | 44.1 | 30.2 | 16.5 | 9.2 |
| ESP | OECD | 50.8 | 19,686 | 42.4 | 22.3 | 35.4 |  | 19,601 | 48.7 | 21.8 | 29.5 |  | 10,082 | 70.5 | 12.4 | 17.1 |  | 1,446 | 83.8 | 6.9 | 9.3 |  |
| HUN | OECD | 13.2 | 5,257 | 13.1 | 61.1 | 25.8 |  | 5,069 | 14.8 | 66.5 | 18.6 |  | 2,654 | 30.7 | 48.2 | 21.1 |  | 199 | 59.1 | 16.4 | 24.5 |  |
| ITA | OECD | 81.9 | 26,488 | 37.1 | 45.4 | 17.5 |  | 32,912 | 51.2 | 36 | 12.8 |  | 20,111 | 72.9 | 18.8 | 8.2 |  | 2,434 | 82.2 | 11 | 6.8 |  |
| KOR | OECD | 69.7 | 27,275 | 1.2 | 24.3 | 74.5 |  | 31,036 | 15.1 | 42.6 | 42.3 |  | 11,196 | 50.1 | 30 | 19.9 |  | 198 | 64.4 | 18.4 | 17.2 |  |
| LTU | OECD | 4.6 | 1,799 | 20.6 | 48.4 | 31 |  | 1,891 | 6.4 | 67.2 | 26.4 |  | 864 | 28.3 | 50.3 | 21.4 |  | 85 | 57.6 | 24.7 | 17.6 |  |
| NZL | OECD | 20.8 | 8,019 | 21 | 35.2 | 29.9 | 13.8 | 8,214 | 29.1 | 33 | 25.8 | 12.1 | 4,114 | 36.8 | 27.8 | 18.1 | 17.4 | 403 | 36.4 | 25.8 | 13.8 | 24 |
| POL | OECD | 75 | 31,450 | 7.9 | 60 | 24.2 | 7.9 | 30,592 | 13.6 | 69.7 | 12.4 | 4.3 | 12,024 | 34.3 | 49.5 | 14 | 2.1 | 896 | 58.3 | 26.1 | 13 | 2.6 |
| SVK | OECD | 7.7 | 3,505 | 6.6 | 67.5 | 25.9 |  | 2,848 | 7.2 | 75.1 | 17.7 |  | 1,212 | 11.9 | 70.9 | 17.2 |  | 87 | 20.3 | 63.4 | 16.2 |  |
| SWE | OECD | 14 | 5,351 | 11.5 | 50.8 | 34.2 | 3.5 | 4,988 | 16.1 | 58.2 | 24.6 | 1.1 | 3,287 | 32.8 | 46.1 | 19.8 | 1.2 | 361 | 48 | 36.2 | 13.9 | 2 |
| TUR | OECD | 45.6 | 24,380 | 44.5 | 29.6 | 24.5 | 1.5 | 15,743 | 65.3 | 19.2 | 14.3 | 1.2 | 5,202 | 82 | 7.4 | 8.7 | 1.9 | 313 | 86.7 | 5.2 | 6.1 | 2 |
| USA | OECD | 215.8 | 87,344 | 11.1 | 48.1 | 40.8 |  | 81,833 | 12.6 | 49 | 38.4 |  | 42,130 | 13.4 | 46.3 | 40.2 |  | 4,452 | 21.8 | 45.6 | 32.7 |  |
| **Average** | | **29.3** | **11,730** | **18.1** | **45.3** | **28.1** | **3** | **11,346** | **22.2** | **47.4** | **22.8** | **1.9** | **5,646** | **35** | **38.7** | **18.9** | **1.8** | **573** | **46.8** | **29.1** | **15.8** | **2.8** |

Table A.3. Distribution of educational attainment by country, source, and age for men

| **Country** | **Source** | **Total Person-Years** | **Age 25-44** | | | | | **Age 45-64** | | | | | **Age 65-84** | | | | | **Age 85+** | | | | |
| --- | --- | --- | --- | --- | --- | --- | --- | --- | --- | --- | --- | --- | --- | --- | --- | --- | --- | --- | --- | --- | --- | --- |
|  |  | **(1,000,000s)** | **Person-Years** | **Low** | **Middle** | **High** | **Missing** | **Person-Years** | **Low** | **Middle** | **High** | **Missing** | **Person-Years** | **Low** | **Middle** | **High** | **Missing** | **Person-Years** | **Low** | **Middle** | **High** | **Missing** |
|  |  |  | **(1000s)** | **(%)** | **(%)** | **(%)** | **(%)** | **(1000s)** | **(%)** | **(%)** | **(%)** | **(%)** | **(1000s)** | **(%)** | **(%)** | **(%)** | **(%)** | **(1000s)** | **(%)** | **(%)** | **(%)** | **(%)** |
| AUS | OECD | 8.4 | 3,438 | 9.9 | 42.7 | 38.8 | 8.5 | 3,028 | 27.6 | 39.4 | 23.2 | 9.9 | 1,653 | 46.3 | 24.8 | 11.5 | 17.3 | 303 | 50.5 | 15.8 | 4.2 | 30 |
| CAN | OECD | 46.3 | 14,435 | 7.7 | 29.2 | 63.2 |  | 19,942 | 12.8 | 39.1 | 48.1 |  | 10,299 | 30.8 | 37.3 | 31.8 |  | 1,612 | 48.7 | 32.9 | 18.4 |  |
| DNK | OECD | 10.1 | 3,519 | 14 | 35.8 | 47.6 | 2.6 | 3,750 | 22.9 | 41.3 | 34.4 | 1.4 | 2,464 | 43.3 | 35.2 | 19.7 | 1.8 | 394 | 57.3 | 19.3 | 9 | 14 |
| ESP | OECD | 54.4 | 19,414 | 30.9 | 21.1 | 48 |  | 19,890 | 47.9 | 21.6 | 30.5 |  | 12,262 | 81 | 9.6 | 9.4 |  | 2,857 | 90.8 | 5.1 | 4.1 |  |
| HUN | OECD | 15.2 | 5,069 | 11.6 | 50.7 | 37.7 |  | 5,491 | 20.7 | 56.4 | 22.9 |  | 4,144 | 48.5 | 37.9 | 13.7 |  | 529 | 80.1 | 13.1 | 6.9 |  |
| ITA | OECD | 91.1 | 26,719 | 29.3 | 44.7 | 26 |  | 34,418 | 50.2 | 36.2 | 13.6 |  | 24,606 | 81.6 | 13.5 | 4.9 |  | 5,335 | 89.5 | 8 | 2.5 |  |
| KOR | OECD | 71.7 | 25,894 | 1.1 | 26.9 | 72 |  | 31,036 | 24.7 | 47.5 | 27.8 |  | 14,264 | 78.4 | 15.8 | 5.8 |  | 472 | 92.1 | 6.1 | 1.8 |  |
| LTU | OECD | 5.8 | 1,794 | 13.8 | 41.5 | 44.8 |  | 2,208 | 4 | 56.3 | 39.7 |  | 1,572 | 32.5 | 48.9 | 18.5 |  | 266 | 68.1 | 21.4 | 10.5 |  |
| NZL | OECD | 22.8 | 8,708 | 18.2 | 30 | 40.6 | 11.2 | 8,854 | 31.6 | 27 | 30 | 11.4 | 4,549 | 45.4 | 17.8 | 18.1 | 18.8 | 703 | 44.8 | 17.1 | 10.3 | 28 |
| POL | OECD | 84 | 30,704 | 5.2 | 49.2 | 36.8 | 8.8 | 32,534 | 14.7 | 65.5 | 16 | 3.9 | 18,224 | 48.3 | 40.8 | 9 | 1.9 | 2,586 | 75.7 | 16.5 | 3.5 | 4 |
| SVK | OECD | 8.3 | 3,335 | 5.7 | 55.1 | 39.2 |  | 2,969 | 11.8 | 69.5 | 18.8 |  | 1,795 | 31.8 | 58.2 | 10 |  | 224 | 57.4 | 37.9 | 4.7 |  |
| SWE | OECD | 14.3 | 5,108 | 8.6 | 40.6 | 48.4 | 2.4 | 4,890 | 11.8 | 50.6 | 36.8 | 0.8 | 3,580 | 29.9 | 43.3 | 25.4 | 1.4 | 676 | 56 | 30.1 | 11.5 | 2 |
| TUR | OECD | 46.6 | 23,795 | 56.2 | 21.4 | 21.4 | 1.1 | 15,702 | 79.8 | 11.4 | 7.4 | 1.4 | 6,438 | 91.4 | 3.8 | 2.6 | 2.2 | 679 | 91.9 | 3 | 1.3 | 4 |
| USA | OECD | 230.6 | 86,342 | 8.6 | 40.8 | 50.6 |  | 85,953 | 10.6 | 46.8 | 42.6 |  | 50,109 | 14.1 | 53.1 | 32.9 |  | 8,156 | 23.6 | 56.5 | 19.9 |  |
| Average | | 30.6 | 86,342 | 9 | 41 | 51 |  | 85,953 | 11 | 47 | 43 |  | 50,109 | 14 | 53 | 33 |  | 8,156 | 24 | 56 | 20 |  |

Table A4. Used correction factors (ratios of CPS deaths to death certificates deaths)

*Source:* classification ratios are obtained from Rostron et al. (2010) Table 4. OECD classification of US educational attainment is based on ISCED 2011.

### A.3. Calculated statistics

#### Life expectancy

Abridged life tables are used to calculate period life expectancy using the Chiang method (Chiang, 1984_[25]_). Pooling observations in 5-year age groups is advantageous as it leads to larger sample sizes, thereby decreasing volatility of mortality rates. Volatile mortality rates are likely to arise in higher age groups with lower exposure levels as small changes in the number of deaths results in large changes in mortality rates.

#### Age-standardised mortality rates

This paper relies on age-standardised mortality rates (ASMR) to account for individual country-level variations in population structures over time and to control for age as a confounder of the education-longevity relationship (Mackenbach et al., 2015_[26]_). Mortality rates are adjusted directly using the 2010 OECD standard population.

#### Absolute and relative gaps

Gaps are simple pairwise comparisons of longevity between two groups. In life expectancy estimates, the absolute (relative) gap refers to the difference (ratio) in life expectancy between the high and low education groups:

$$Absolute Gap=LifeExpectancy_{High}-LifeExpectancy_{Low}$$

$$Relative Gap=\frac{LifeExpectancy_{High}}{LifeExpectancy_{Low}}$$

In principle, other pairwise comparisons between education groups could also be conducted. We only present comparisons of low and high education groups for the sake of brevity.

When applied to standardised mortality rates, the same concepts are referred to as rate difference (RD) and rate ratio (RR), for absolute and relative inequality measurement, respectively.

$$Rate Difference={MortalityRate}_{Low}-{MortalityRate}_{High}$$

$$Rate Ratio=\frac{MortalityRate_{Low}}{MortalityRate_{High}}$$

#### Slope and relative indices of Inequality

The slope and relative indices of Inequality (SII; RII) are used to account for the entire education distribution and to provide an overall assessment of inequality across education groups. For the SII, this is accomplished by regressing the longevity outcome on a fraction-ranked education weighted by the education distribution, using an ordinary least squares regression to measure absolute inequality and a logistic regression to measure relative inequality (Moreno-Betancur et al., 2015_[27]_). While Poisson distributions may, strictly speaking, be better to model mortality rates according to some authors, we have used the normal distribution as in the previous analysis (Murtin et al., 2017_[1]_), and checked that the differences in results between the two methods are minimal. Thus, the SII is:

$$y=g_{\alpha}\left( x \right)=y_{0}+\alpha x$$

$$SII=\alpha=g_{\alpha}\left( 1 \right)-g_{\alpha}(0)$$

where α is the magnitude and sign of the linear association between *x* and *y*, the education and longevity variables, respectively, when education is fraction-ranked between 0 and 1. SII values greater than zero indicate that the more educated groups have greater longevity than less educated groups. Conversely, if the SII is less than zero, the less educated have greater longevity. A vakue of the SII of zero indicates no inequalities in longevity between education groups. For standardised mortality rates, the absolute value of the SII is presented to maintain consistency in results interpretation.

The RII is calculated as:

$$y=f_{\beta}\left( x \right)=y_{0}exp(\beta x)$$

$$RII=\exp\left( \beta\right)=\frac{f_{\beta}(1)}{f_{\beta}(0)}$$

where *exp(β)* is the magnitude of the linear association between *x* and *y*, the education and longevity variables, respectively, when education is fraction-ranked between 0 and 1. An RII estimate equal to one indicates no relative advantage between predicted low and high education groups. If the RII is greater than one, higher education is associated with relatively greater longevity and when RII is less than one, higher education is associated with relatively lesser longevity. For example, a RII of 1.1 indicates that the highest education group has 10%, or 1.1 times, greater longevity than the predicted lowest education group. For standardised mortality rates, the inverse of the RII is presented to maintain consistency in results interpretation.

#### Decomposition of life expectancy gaps

We use the Arriaga method to decompose cause- and age-specific contributions to life expectancy gaps (Arriaga, 1984_[29]_). The gap in life expectancy is decomposed into direct, indirect and interaction effects, which sum to the total effect, namely the absolute life expectancy gap in years. We follow the two-step calculation approach of (Auger et al., 2014_[30]_). The age-group total contribution to the life expectancy gap is the sum of the direct, indirect, and interaction effect:

$$nC_{x}=\left[ \frac{l_{x}^{Low}}{l_{25}}\times\left( \frac{nL_{x}^{High}}{l_{x}^{High}}-\frac{nL_{x}^{Low}}{l_{x}^{Low}} \right) \right]+\left[ \frac{T_{x+n}^{High}}{l_{25}}\times\left( \frac{l_{x}^{Low}}{l_{x}^{High}}-\frac{nl_{x+n}^{Low}}{l_{x+n}^{Low}} \right) \right]$$

where *High* and *Low* represent the high and low education groups, *nC_x_* is the total contribution between ages *x* and *x+n*, *l_x_* is the remaining cohort size at age *x*, *nL_x_* is the person-years lived between ages *x* and *x+n*, *l_25_* is the cohort size at age 25, and *T_x+n_* is the person-years lived above age *x+n*. The cause-specific contribution to the life expectancy gap is then:

$$nC_{x}^{i}=nC_{x}\times\left[ \frac{\left( n{R_{x}^{i}}^{High}\times nM_{x}^{High} \right)-\left( n{R_{x}^{i}}^{Low}\times nM_{x}^{Low} \right)}{nM_{x}^{High}\times{nM}_{x}^{Low}} \right]$$

where *nR_x_i* is the proportion of deaths attributable to cause *i* at age *x* and *nM_x_* is the all-cause mortality rate at age *x*.

Appendix B. ADDITIONAL RESULTS

Table B.1. Deaths of despair age-standardised mortality rates by country, sex, age-group, and education

| **Country** | **Females** | | | | | | **Males** | | | | | |
| --- | --- | --- | --- | --- | --- | --- | --- | --- | --- | --- | --- | --- |
|  | **25-64** | | | **65-89** | | | **25-64** | | | **65-89** | | |
|  | **Low** | **Middle** | **High** | **Low** | **Middle** | **High** | **Low** | **Middle** | **High** | **Low** | **Middle** | **High** |
| AUS | 28 | 19 | 10 | 11 | 15 | 16 | 92 | 47 | 20 | 51 | 39 | 30 |
| CAN | 35 | 21 | 12 | 10 | 16 | 14 | 87 | 59 | 30 | 74 | 51 | 35 |
| DNK | 26 | 11 | 8 | 24 | 31 | 30 | 48 | 23 | 16 | 74 | 58 | 54 |
| ESP | 7 | 8 | 3 | 9 | 10 | 7 | 25 | 24 | 7 | 44 | 31 | 15 |
| HUN | 45 | 23 | 12 | 57 | 43 | 33 | 206 | 91 | 33 | 324 | 164 | 100 |
| ITA | 4 | 4 | 3 | 6 | 6 | 6 | 18 | 12 | 9 | 27 | 17 | 13 |
| KOR | 152 | 31 | 13 | 28 | 19 | 19 | 340 | 90 | 31 | 149 | 94 | 56 |
| LTU | 16 | 11 | 5 | 13 | 11 | 9 | 82 | 57 | 27 | 87 | 56 | 28 |
| NZL | 14 | 11 | 7 | 6 | 8 | 6 | 37 | 25 | 16 | 29 | 25 | 25 |
| POL | 9 | 5 | 3 | 7 | 6 | 5 | 78 | 35 | 12 | 54 | 37 | 12 |
| SVK | 43 | 21 | 9 | 31 | 17 | 17 | 202 | 72 | 34 | 241 | 89 | 40 |
| SWE | 42 | 20 | 10 | 27 | 26 | 17 | 94 | 49 | 22 | 76 | 66 | 40 |
| TUR | 1 | 1 | 1 | 2 | 2 | 8 | 5 | 5 | 4 | 5 | 6 | 5 |
| USA | 62 | 54 | 20 | 15 | 20 | 17 | 147 | 129 | 44 | 84 | 87 | 49 |
| **Average** | **35** | **17** | **8** | **18** | **17** | **15** | **104** | **51** | **22** | **94** | **59** | **36** |

Note: Countries are reported in International Organization for Standardization (ISO) three-letter codes. Education is classified according to the 2011 International Standard Classification of Education (ISCED-2011) into low (lower secondary education and below, ISCED 0-2), medium (upper-secondary, ISCED 3-4), and high education (higher than upper-secondary, ISCED 5-8). Mortality rates are standardised using the OECD 2010 standard population. Deaths of Despair: Suicide (X60-X84, Y87.0), Alcohol-Related Deaths (E24.4, F10, G31.2, G62.1, G72.1, I42.6, K29.2, K70, K85.2, K86.0, O35.4, P04.3, Q86.0, R78.0, X45, Y15), and Drug-Related Deaths (F11-16, X40-44, Y10-14).

Table B.2. Rate difference, rate ratio, slope index of inequality, and relative index of inequality in age-standardised mortality rates for deaths of despair by country, age, and sex

| **Country** | **Females** | | | | | | | | **Males** | | | | | | | |
| --- | --- | --- | --- | --- | --- | --- | --- | --- | --- | --- | --- | --- | --- | --- | --- | --- |
|  | **25-64** | | | | **65-89** | | | | **25-64** | | | | **65-89** | | | |
|  | **SII** | **RD** | **RII** | **RR** | **SII** | **RD** | **RII** | **RR** | **SII** | **RD** | **RII** | **RR** | **SII** | **RD** | **RII** | **RR** |
| AUS | 24 | 18 | 3.797 | 2.754 | -8 | -5 | 0.569 | 0.698 | 89 | 72 | 6.891 | 4.555 | 29 | 21 | 1.988 | 1.718 |
| CAN | 27 | 23 | 4.843 | 2.933 | -6 | -5 | 0.683 | 0.675 | 74 | 57 | 4.52 | 2.879 | 55 | 39 | 3.002 | 2.125 |
| DNK | 22 | 19 | 9.208 | 3.467 | -10 | -6 | 0.706 | 0.803 | 41 | 33 | 6.201 | 3.088 | 30 | 21 | 1.64 | 1.39 |
| ESP | 6 | 3 | 2.415 | 1.953 | 2 | 2 | 1.238 | 1.352 | 27 | 18 | 3.887 | 3.38 | 46 | 29 | 4.306 | 2.955 |
| HUN | 38 | 33 | 6.141 | 3.653 | 32 | 23 | 1.977 | 1.701 | 196 | 173 | 8.526 | 6.225 | 300 | 224 | 5.191 | 3.249 |
| ITA | 1 | 1 | 1.335 | 1.25 | 0 | 0 | 0.949 | 0.987 | 13 | 9 | 2.618 | 2.066 | 22 | 14 | 2.844 | 2.031 |
| KOR | 129 | 139 | 357.616 | 12.015 | 17 | 9 | 2.112 | 1.445 | 252 | 309 | 351.081 | 10.998 | 140 | 93 | 3.711 | 2.665 |
| LTU | 13 | 11 | 4.811 | 3.23 | 6 | 4 | 1.632 | 1.508 | 69 | 55 | 3.621 | 3 | 76 | 59 | 3.563 | 3.114 |
| NZL | 10 | 7 | 2.713 | 1.956 | -1 | 0 | 0.916 | 1.054 | 30 | 21 | 3.26 | 2.268 | 7 | 4 | 1.302 | 1.163 |
| POL | 6 | 6 | 4.095 | 2.879 | 3 | 3 | 1.637 | 1.578 | 71 | 66 | 7.675 | 6.496 | 51 | 42 | 3.292 | 4.523 |
| SVK | 34 | 34 | 7.647 | 4.991 | 22 | 14 | 3.118 | 1.805 | 140 | 168 | 12.494 | 5.974 | 209 | 201 | 10.429 | 5.989 |
| SWE | 33 | 32 | 8.614 | 4.258 | 14 | 11 | 1.758 | 1.622 | 83 | 72 | 6.395 | 4.239 | 46 | 36 | 1.966 | 1.916 |
| TUR | 1 | 1 | 1.888 | 1.665 | -5 | -6 | 0.177 | 0.248 | 2 | 2 | 1.675 | 1.465 | 0 | 0 | 1.023 | 1.099 |
| USA | 69 | 43 | 4.843 | 3.18 | 1 | -2 | 1.039 | 0.874 | 163 | 103 | 4.226 | 3.345 | 64 | 35 | 2.254 | 1.711 |
| **Average** | **29** | **26** | **29.998** | **3.584** | **5** | **3** | **1.322** | **1.168** | **89** | **83** | **30.219** | **4.284** | **77** | **59** | **3.322** | **2.546** |

Note: SII, slope index of inequality; RII, relative index of inequality; RD, rate difference; RR, rate ratio.

Mortality rates are standardised using the OECD 2010 standard population. Deaths of Despair: Suicide (X60-X84, Y87.0), Alcohol-Related Deaths (E24.4, F10, G31.2, G62.1, G72.1, I42.6, K29.2, K70, K85.2, K86.0, O35.4, P04.3, Q86.0, R78.0, X45, Y15), and Drug-Related Deaths (F11-16, X40-44, Y10-14)

Figure B.1. Decomposition of deaths of despair contribution to the life expectancy gap (in years) by country, sex, and age (high vs low education).


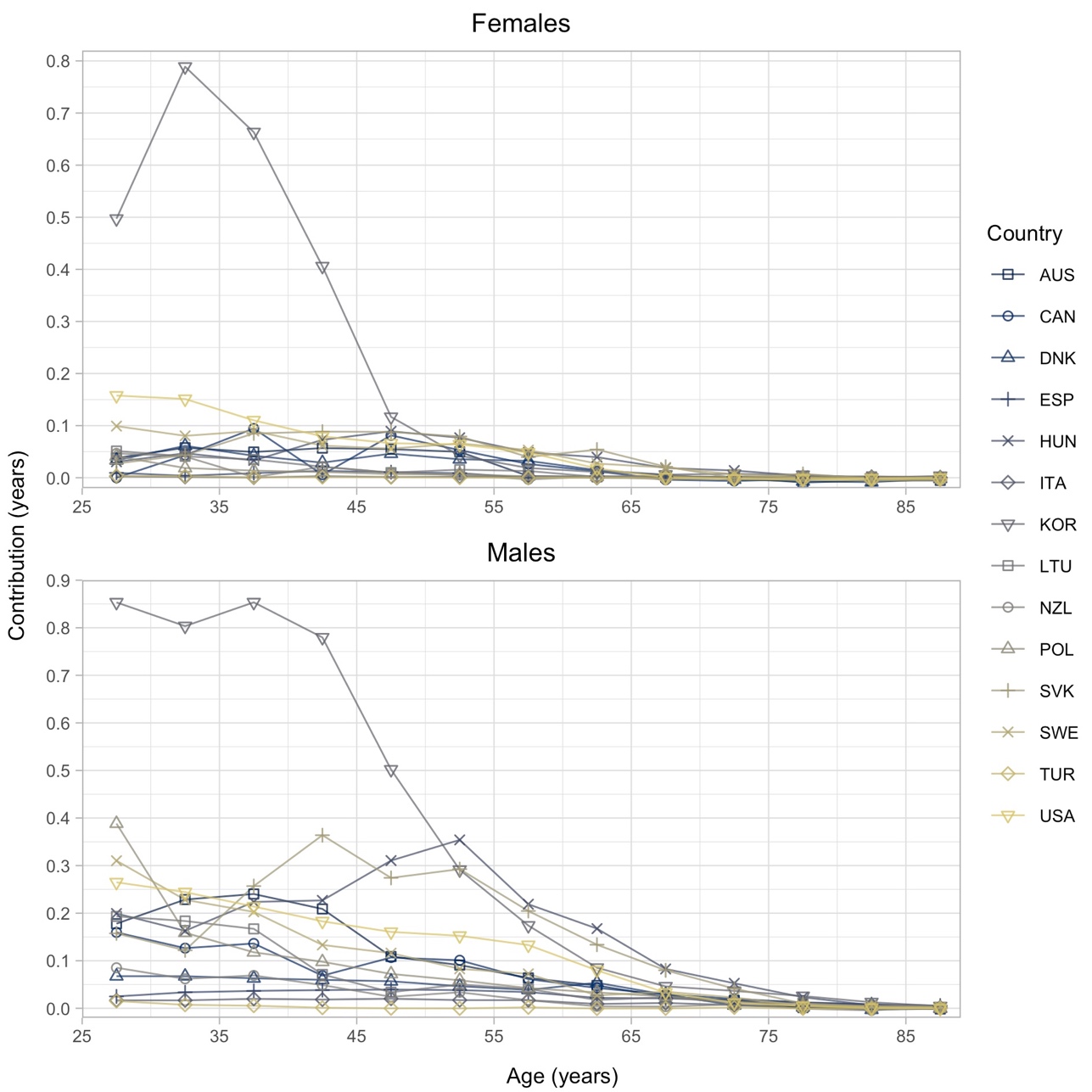


Notes: Countries are reported in International Organization for Standardization (ISO) three-letter codes. Education is classified according to the 2011 International Standard Classification of Education (ISCED-2011) into low (primary education and below, ISCED 0-2), medium (lower- and upper-secondary, ISCED 3-4), and high education (higher than upper-secondary, ISCED 5-8). Deaths of Despair: Suicide (X60-X84, Y87.0), Alcohol-Related Deaths (E24.4, F10, G31.2, G62.1, G72.1, I42.6, K29.2, K70, K85.2, K86.0, O35.4, P04.3, Q86.0, R78.0, X45, Y15), and Drug-Related Deaths (F11-16, X40-44, Y10-14).

Figure B.2. Decomposition of deaths of despair contribution to the life expectancy gap (in years) between groups by country, sex, and age (high vs low education, excluding Korea)


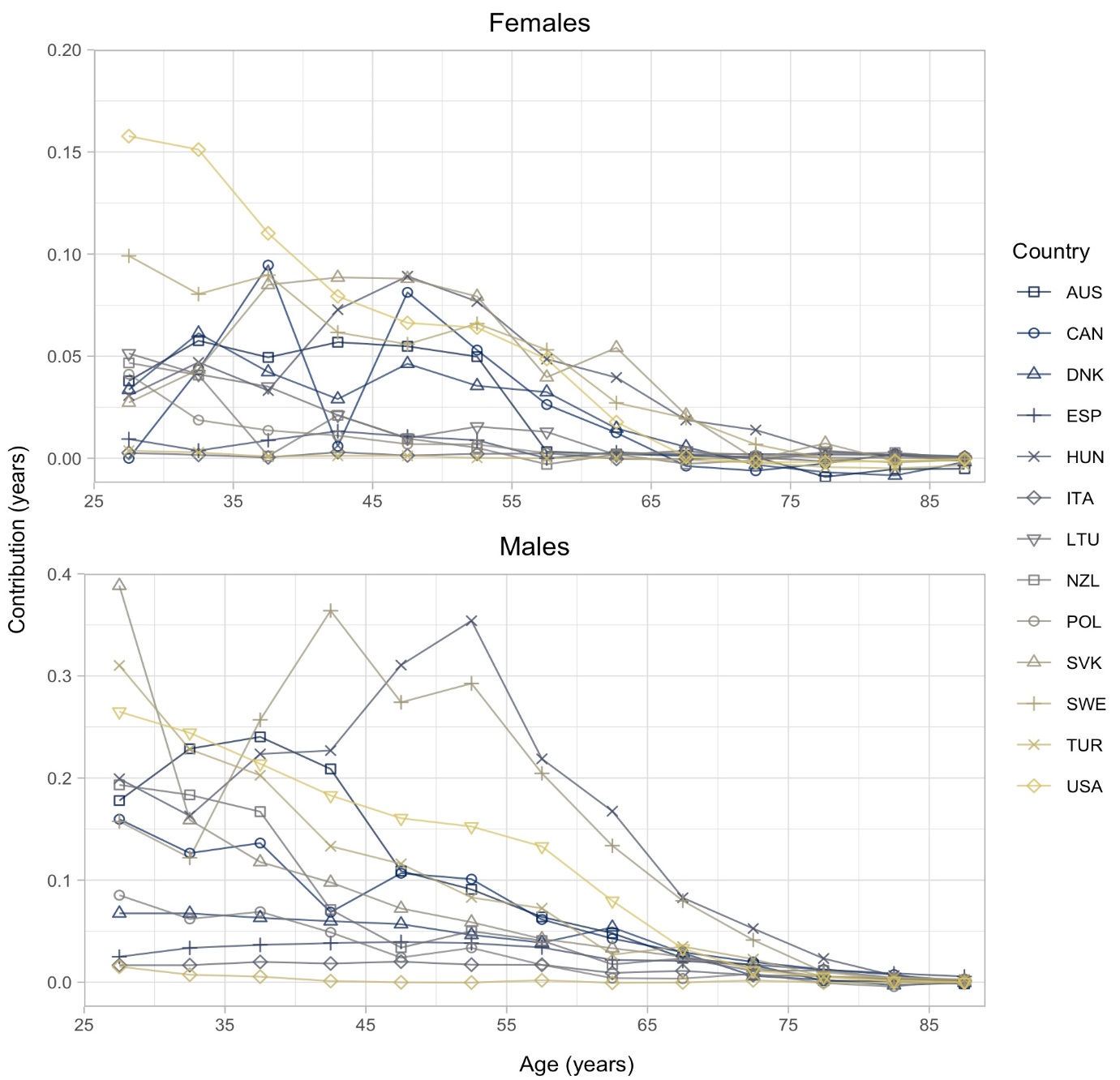


Notes: Countries are reported in International Organization for Standardization (ISO) three-letter codes. Education is classified according to the 2011 International Standard Classification of Education (ISCED-2011) into low (primary education and below, ISCED 0-2), medium (lower- and upper-secondary, ISCED 3-4), and high education (higher than upper-secondary, ISCED 5-8). Deaths of Despair: Suicide (X60-X84, Y87.0), Alcohol-Related Deaths (E24.4, F10, G31.2, G62.1, G72.1, I42.6, K29.2, K70, K85.2, K86.0, O35.4, P04.3, Q86.0, R78.0, X45, Y15), and Drug-Related Deaths (F11-16, X40-44, Y10-14).

Figure B.3. Decomposition of deaths of despair contribution to the life expectancy gap (in years) by country, sex, and age around 2016 (middle vs high education)

*
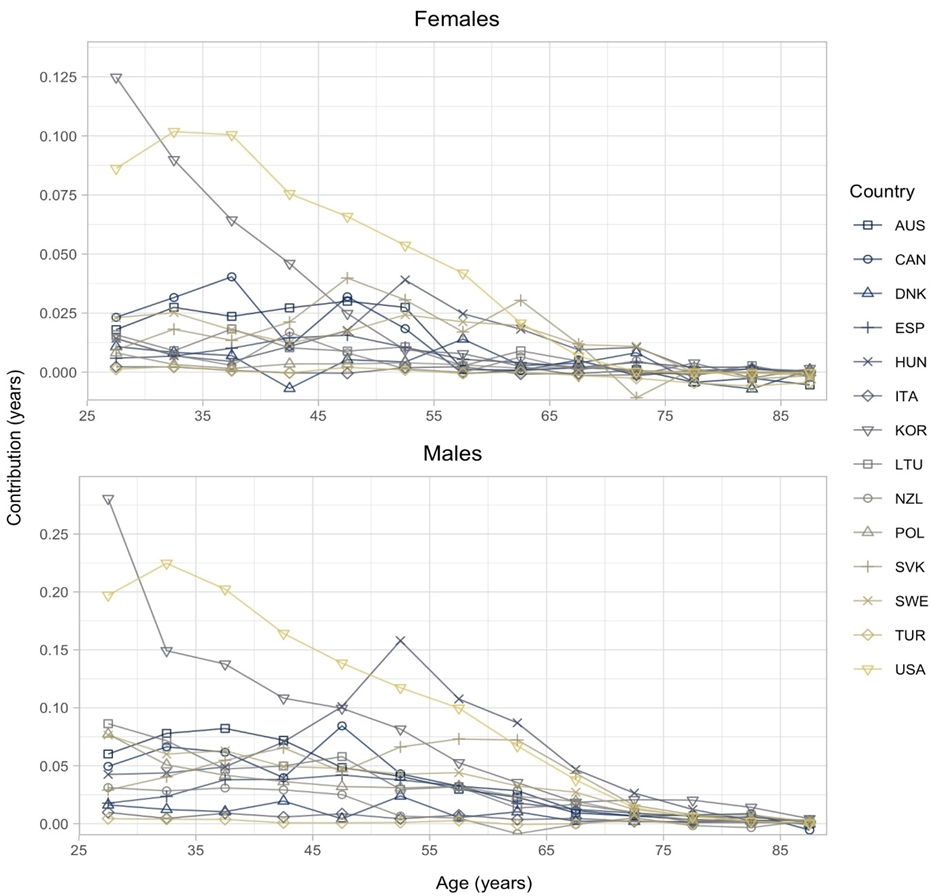
*

Notes: Countries are reported in International Organization for Standardization (ISO) three-letter codes. Education is classified according to the 2011 International Standard Classification of Education (ISCED-2011) into low (primary education and below, ISCED 0-2), medium (lower- and upper-secondary, ISCED 3-4), and high education (higher than upper-secondary, ISCED 5-8). Deaths of Despair: Suicide (X60-X84, Y87.0), Alcohol-Related Deaths (E24.4, F10, G31.2, G62.1, G72.1, I42.6, K29.2, K70, K85.2, K86.0, O35.4, P04.3, Q86.0, R78.0, X45, Y15), and Drug-Related Deaths (F11-16, X40-44, Y10-14).

Figure B.4. Slope and relative indices of inequality in deaths of despair age-standardised mortality rates by country, sex, and age group around 2016


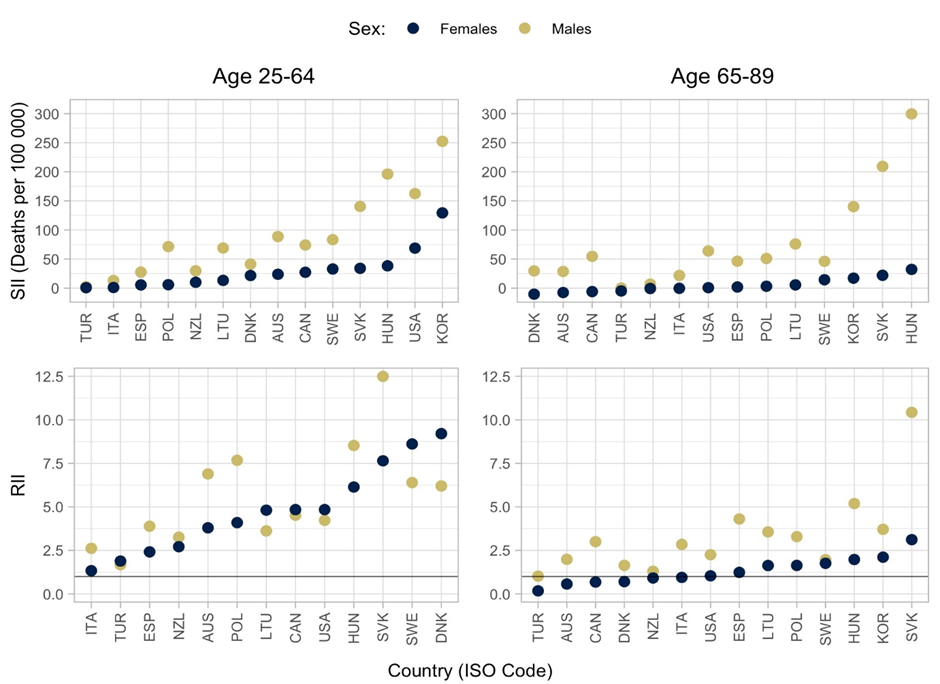
Note: SII, slope index of inequality; RII, relative index of inequality. Countries are reported in International Organization for Standardization (ISO) three-letter codes. Mortality rates are standardised using the OECD 2010 standard population. Deaths of Despair: Suicide (X60-X84, Y87.0), Alcohol-Related Deaths (E24.4, F10, G31.2, G62.1, G72.1, I42.6, K29.2, K70, K85.2, K86.0, O35.4, P04.3, Q86.0, R78.0, X45, Y15), and Drug-Related Deaths (F11-16, X40-44, Y10-14).

Figure B.5. Deaths of despair relative contribution to rate differences in age-standardised mortality rates by country, sex, and age-group


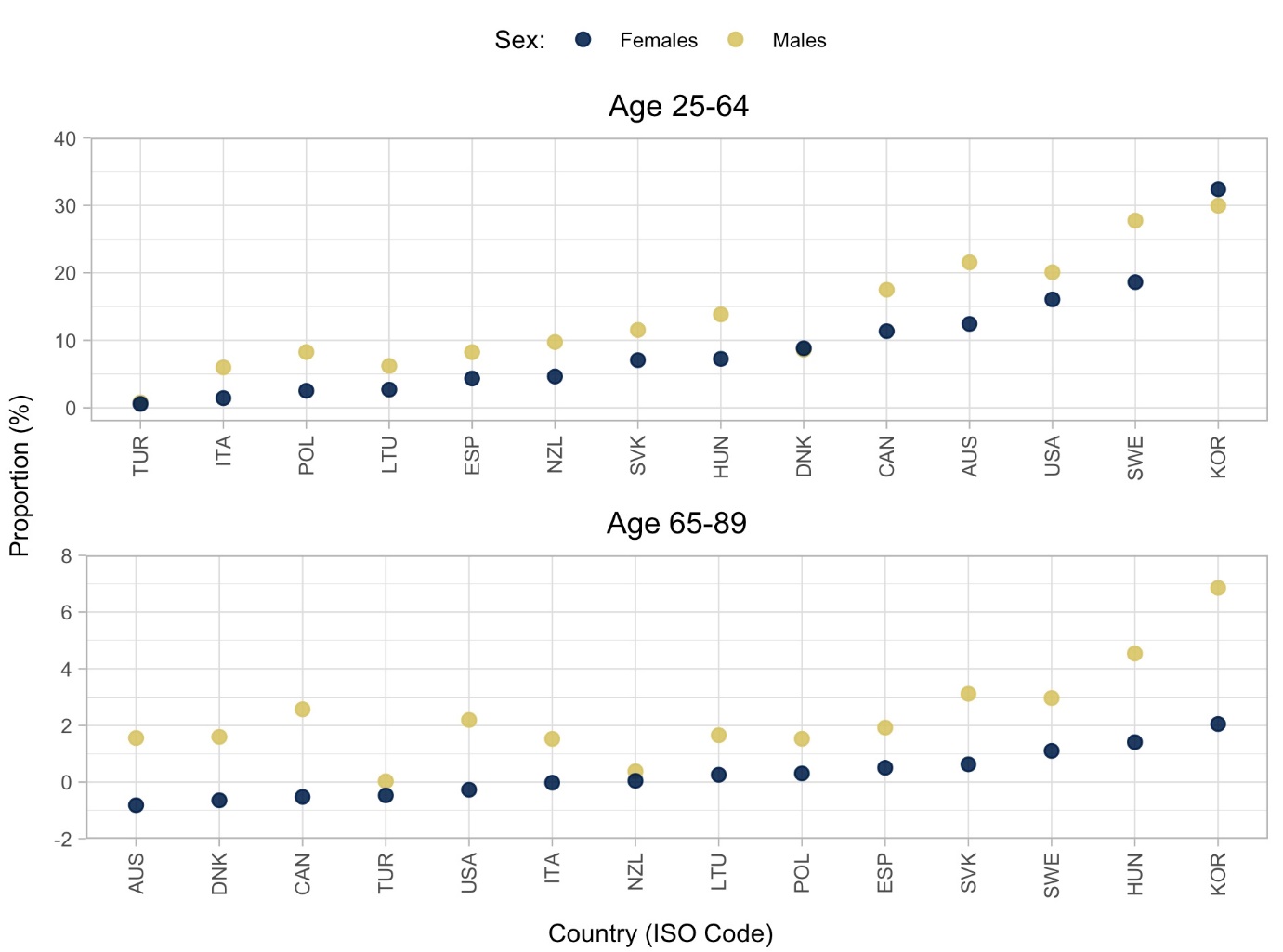


Note: Countries are reported in International Organization for Standardization (ISO) three-letter codes. Education is classified according to the 2011 International Standard Classification of Education (ISCED-2011) into low (lower secondary education and below, ISCED 0-2), medium (upper-secondary, ISCED 3-4), and high education (higher than upper-secondary, ISCED 5-8). Mortality rates are standardised using the OECD 2010 standard population. Deaths of Despair: Suicide (X60-X84, Y87.0), Alcohol-Related Deaths (E24.4, F10, G31.2, G62.1, G72.1, I42.6, K29.2, K70, K85.2, K86.0, O35.4, P04.3, Q86.0, R78.0, X45, Y15), and Drug-Related Deaths (F11-16, X40-44, Y10-14).

**Table B.3. Decomposition of deaths of despair contribution to the total life expectancy gap (in years and percent) by country, sex, and age group.**

|  |  | | | | | | |  |  |  |  |  |  |  |  |
| --- | --- | --- | --- | --- | --- | --- | --- | --- | --- | --- | --- | --- | --- | --- | --- |
| **Country** | **High vs Low Education** | | | | | | |  | **High vs Middle Education** | | | | | | |
|  | **Females** | | |  | **Males** | | |  | **Females** | | |  | **Males** | | |
|  | **25-44** | **45-64** | **65+** |  | **25-44** | **45-64** | **65+** |  | **25-44** | **45-64** | **65+** |  | **25-44** | **45-64** | **65+** |
| AUS | 0.20 | 0.11 | -0.02 |  | 0.86 | 0.31 | 0.04 |  | 0.10 | 0.06 | -0.01 |  | 0.29 | 0.14 | 0.02 |
| *% LE Gap (all ages)* | *4.7* | *2.6* | *-0.5* |  | *11.1* | *4.0* | *0.5* |  | *6.1* | *3.8* | *-0.7* |  | *9.0* | *4.4* | *0.6* |
| CAN | 0.14 | 0.17 | -0.01 |  | 0.49 | 0.31 | 0.07 |  | 0.11 | 0.05 | 0.01 |  | 0.22 | 0.19 | 0.03 |
| *% LE Gap (all ages)* | *2.8* | *3.3* | *-0.2* |  | *6.9* | *4.4* | *1.0* |  | *4.4* | *2.1* | *0.2* |  | *5.8* | *5.0* | *0.8* |
| DNK | 0.17 | 0.13 | -0.02 |  | 0.26 | 0.20 | 0.03 |  | 0.02 | 0.03 | 0.00 |  | 0.06 | 0.04 | 0.01 |
| *% LE Gap (all ages)* | *3.2* | *2.5* | *-0.3* |  | *3.9* | *2.9* | *0.5* |  | *1.1* | *1.4* | *0.0* |  | *1.8* | *1.4* | *0.3* |
| ESP | 0.04 | 0.02 | 0.01 |  | 0.13 | 0.13 | 0.07 |  | 0.04 | 0.04 | 0.01 |  | 0.12 | 0.13 | 0.04 |
| *% LE Gap (all ages)* | *1.0* | *0.6* | *0.2* |  | *2.0* | *2.0* | *1.0* |  | *1.2* | *1.2* | *0.3* |  | *1.8* | *2.0* | *0.6* |
| HUN | 0.18 | 0.25 | 0.04 |  | 0.81 | 1.05 | 0.17 |  | 0.04 | 0.10 | 0.02 |  | 0.21 | 0.45 | 0.09 |
| *% LE Gap (all ages)* | *2.2* | *3.1* | *0.4* |  | *5.4* | *7.0* | *1.1* |  | *1.8* | *4.9* | *0.9* |  | *4.0* | *8.7* | *1.8* |
| ITA | 0.01 | 0.01 | 0.00 |  | 0.07 | 0.06 | 0.03 |  | 0.00 | 0.00 | 0.00 |  | 0.03 | 0.02 | 0.01 |
| *% LE Gap (all ages)* | *0.3* | *0.2* | *0.0* |  | *1.8* | *1.6* | *0.7* |  | *0.7* | *0.4* | *0.1* |  | *1.8* | *1.4* | *0.6* |
| KOR | 2.36 | 0.19 | 0.02 |  | 3.29 | 1.05 | 0.13 |  | 0.33 | 0.04 | 0.00 |  | 0.68 | 0.27 | 0.08 |
| *% LE Gap (all ages)* | *27.1* | *2.2* | *0.3* |  | *21.4* | *6.9* | *0.8* |  | *20.6* | *2.8* | *0.0* |  | *16.4* | *6.5* | *1.9* |
| LTU | 0.15 | 0.04 | 0.01 |  | 0.62 | 0.14 | 0.05 |  | 0.05 | 0.03 | 0.01 |  | 0.25 | 0.13 | 0.04 |
| *% LE Gap (all ages)* | *1.7* | *0.5* | *0.1* |  | *5.1* | *1.2* | *0.4* |  | *1.7* | *1.0* | *0.2* |  | *3.7* | *1.9* | *0.5* |
| NZL | 0.11 | 0.01 | 0.00 |  | 0.27 | 0.08 | 0.01 |  | 0.04 | 0.02 | 0.01 |  | 0.12 | 0.03 | 0.00 |
| *% LE Gap (all ages)* | *2.8* | *0.4* | *0.0* |  | *5.3* | *1.6* | *0.2* |  | *2.9* | *1.1* | *0.4* |  | *5.5* | *1.3* | *0.1* |
| POL | 0.08 | 0.02 | 0.01 |  | 0.76 | 0.21 | 0.05 |  | 0.02 | 0.01 | 0.00 |  | 0.21 | 0.12 | 0.03 |
| *% LE Gap (all ages)* | *1.6* | *0.4* | *0.1* |  | *6.2* | *1.7* | *0.4* |  | *0.5* | *0.4* | *0.1* |  | *2.6* | *1.5* | *0.4* |
| SVK | 0.24 | 0.26 | 0.03 |  | 0.90 | 0.91 | 0.13 |  | 0.06 | 0.12 | 0.00 |  | 0.19 | 0.26 | 0.07 |
| *% LE Gap (all ages)* | *2.2* | *2.4* | *0.2* |  | *5.3* | *5.4* | *0.8* |  | *1.6* | *3.1* | *0.0* |  | *4.0* | *5.4* | *1.4* |
| SWE | 0.33 | 0.20 | 0.02 |  | 0.87 | 0.30 | 0.07 |  | 0.08 | 0.08 | 0.02 |  | 0.25 | 0.17 | 0.06 |
| *% LE Gap (all ages)* | *6.3* | *3.9* | *0.4* |  | *14.2* | *4.8* | *1.2* |  | *3.0* | *3.2* | *0.8* |  | *8.5* | *5.7* | *1.9* |
| TUR | 0.01 | 0.00 | -0.02 |  | 0.03 | 0.00 | 0.00 |  | 0.00 | 0.00 | -0.03 |  | 0.01 | 0.00 | 0.00 |
| *% LE Gap (all ages)* | *0.2* | *0.0* | *-0.5* |  | *0.6* | *0.0* | *0.0* |  | *0.3* | *0.2* | *-2.1* |  | *0.5* | *0.1* | *0.1* |
| USA | 0.50 | 0.20 | 0.00 |  | 0.91 | 0.53 | 0.05 |  | 0.36 | 0.18 | 0.01 |  | 0.79 | 0.42 | 0.06 |
| *% LE Gap (all ages)* | *9.5* | *3.7* | *-0.1* |  | *10.8* | *6.3* | *0.6* |  | *9.6* | *4.8* | *0.2* |  | *12.2* | *6.6* | *0.9* |
| **Average (Years)** | **0.33** | **0.12** | **0.00** |  | **0.74** | **0.39** | **0.06** |  | **0.11** | **0.06** | **0.00** |  | **0.28** | **0.19** | **0.04** |
| ***Average (% Total)*** | ***5.36*** | ***2.10*** | ***0.05*** |  | ***7.87*** | ***4.00*** | ***0.70*** |  | ***4.62*** | ***2.49*** | ***0.19*** |  | ***6.38*** | ***4.17*** | ***0.91*** |

Notes: Countries are reported in International Organization for Standardization (ISO) three-letter codes. Education is classified according to the 2011 International Standard Classification of Education (ISCED-2011) into low (primary education and below, ISCED 0-2), medium (lower- and upper-secondary, ISCED 3-4), and high education (higher than upper-secondary, ISCED 5-8). Deaths of Despair: Suicide (X60-X84, Y87.0), Alcohol-Related Deaths (E24.4, F10, G31.2, G62.1, G72.1, I42.6, K29.2, K70, K85.2, K86.0, O35.4, P04.3, Q86.0, R78.0, X45, Y15), and Drug-Related Deaths (F11-16, X40-44, Y10-14).

Figure B.6. Predicted mortality rates for Korea

1. Murtin, F. and C. Lübker (2022), "Educational inequalities in longevity among OECD countries around 2016", *OECD Papers on Well-being and Inequalities*, No. 8, OECD Publishing, Paris, <https://doi.org/10.1787/5faaa751-en>. [↑](#footnote-ref-1)
2. Tjepkema M, Christidis T, Bushnik T, Pinault L. Cohort profile: The Canadian Census Health and Environment Cohorts (CanCHECs). Health Rep. 2019;30(12):18-26. [↑](#footnote-ref-2)
3. Mackenbach, J., et al. (2015). Measuring Educational Inequalities in Mortality Statistics. OECD Statistics Working Papers. Paris. 08. [↑](#footnote-ref-3)
4. Countries corrected from age 85: Korea, Poland, and Lithuania. Countries corrected from age 90: USA, Denmark, and New Zealand. Countries corrected from age 95: Canada, Hungary, and Slovakia. Countries corrected from age 100: Austria, Lithuania, Netherlands, and Turkey. Countries corrected from age 105: Australia, Spain, Italy, and Sweden. [↑](#footnote-ref-4)
